# Multidisciplinary perspectives on artificial intelligence in aging research and education: evolving uses, ethics, and equity considerations in gerontology

**DOI:** 10.1093/geront/gnaf314

**Published:** 2025-12-18

**Authors:** Angela K Perone, Peter M Abadir, Nancy Berlinger, James R Carey, M Aaron Guest, Zachary J Hass, Abigail T Stephan, Bo Xie

**Affiliations:** School of Social Welfare, University of California, Berkeley, California, United States; School of Medicine, Johns Hopkins University, Baltimore, Maryland, United States; The Hastings Center, Garrison, New York, United States; Department of Entomology, University of California, Davis, California, United States; Edson College of Nursing and Health Innovation, Arizona State University, Phoenix, Arizona, United States; School of Nursing, Edwardson School of Industrial Engineering, and Regenstrief Center for Healthcare Engineering, Purdue University, West Lafayette, Indiana, United States; College of Behavioral, Social and Health Sciences, Clemson University, Clemson, South Carolina, United States; School of Nursing, University of Texas at Austin, Austin, Texas, United States; School of Information, University of Texas at Austin, Austin, Texas, United States

**Keywords:** Technology, Interdisciplinary, Training, Geriatrics

## Abstract

Artificial intelligence (AI) models and applications are proliferating rapidly throughout gerontological research and education. Machine learning has catapulted gerontological research in diagnosing and treating age-related health conditions. Students and educators have new tools for customized learning and innovation. Yet many of these developments come with persistent challenges, including bias, inaccuracy, and data security. As in other fields, engagement with AI models in gerontology is often siloed within disciplines. Exploring common opportunities and challenges in this space requires collaboration and conversations across disciplines. To fill this gap, the Gerontological Society of America (GSA)’s Public Policy Advisory Panel convened a multidisciplinary panel discussion of experts from the six GSA member groups and three advisory panels in November 2024 to discuss how AI is shaping various disciplines, and what ethical issues exist within or across disciplines. Several common themes emerged across disciplines: (1) human interaction remains critical to offset AI limitations in human experience, abstract reasoning, creativity, and bias; (2) AI provides opportunities for customized support across disciplines for older adults, care partners, practitioners, researchers, and students; (3) ongoing training is essential to navigate this rapidly evolving landscape; and (4) cross-disciplinary collaboration is needed to address overlapping challenges, limitations, and risks concerning AI.

## Introduction

Artificial Intelligence (AI) is increasingly used in aging research and education to analyze vast amounts of data and identify patterns that may offer insights into aging across the life course (e.g., Bernal et al., 2024; López-Otín, 2023; Ma et al., 2023). By utilizing AI-based systems, researchers, practitioners, and educators can more efficiently study factors like genetics, lifestyle, and social and environmental influences on aging (e.g., Abdallah et al., 2023; Mamoshina et al., 2018). Machine learning techniques may also help predict the onset of age-related diseases and potential interventions (e.g., Alexandru et al., 2024; Bozyel et al., 2024; Coppens et al., 2024). Incorporating AI in aging research and education could potentially revolutionize how we learn about aging and improve the quality of life for older adults.

However, increasing uses of AI have led to many concerns about ethical boundaries and equity while also raising socio-political, socio-cultural, and socio-economic questions about its use in the context of aging. For example, existing literature has documented concerns about AI’s use in disseminating misinformation and disinformation (Cianciulli et al., 2025; Saeidnia et al., 2025), violating privacy and exposing personal or sensitive data (Al-kfairy et al., 2024; Al-Kharusi et al., 2024), facilitating scams (Pietri et al., 2025), engaging in bias and discrimination (Afreen et al., 2025), infringing on intellectual property and copyright (Pappachan et al., 2024), eroding critical thinking (Aboodi, 2025; Szmyd & Mitera, 2024), and adversely affecting the environment through immense energy consumption (Pimenow et al., 2024). These concerns exist across disciplinary boundaries (e.g., Abbonato et al., 2024; Al-kfairy et al., 2024).

While the implications for these technological transformations and ethical dilemmas transcend disciplines, conversations are often siloed in disciplinary spaces (e.g., Hassoun et al., 2022; Vicsek, 2021; Zhau et al., 2022). More opportunities are needed for gerontological scholars to come together and share challenges and opportunities relating to AI and aging in their respective areas of study. We argue that this multidisciplinary approach is not only helpful but that it is essential as emerging technologies in AI rapidly change the landscape of aging.

In response to this growing need, the Gerontological Society of America (GSA)’s Public Policy Advisory Panel organized a 90-minute multidisciplinary session at GSA’s Annual Scientific Meeting on November 14, 2024, addressing the use of AI in aging research and education and subsequent policy implications. GSA is the oldest and largest interdisciplinary organization focused on research, education, and practice in aging (GSA, n.d.). Seven panelists (and one moderator) represented the six member groups of GSA: (1) Biological Sciences, (2) Behavioral and Social Sciences, (3) Health Sciences, (4) Social Research, Policy, and Practice, (5) Academy for Gerontology in Higher Education, and (6) Emerging Scholar and Professional Organization (ESPO), along with the Minority Issues in Aging and the Humanities, Arts, and Culture Gerontology Advisory Panels. Panelists were selected as experts in their discipline by each group chair and members. Prior to the session, organizers asked panelists to consider the following to guide the discussion:

1. How is AI currently shaping research and education in your field? What’s next for AI in your field?
2. What are some ethical issues associated with AI in your field?
  - How are (or should) these issues be addressed?
  - How do these issues impact equity and inclusion in aging and/or for educators/researchers in aging?

This session was one of the most well-attended, with nearly 130 participants, underscoring the hunger for such multidisciplinary collaborations, including cross-field conversations about AI uses and limitations of AI. For example, in biology, AI is used to identify genetic markers of aging, predicting biological age, aiding biomarker discovery, and predicting cellular senescence and mitochondrial decline, thereby addressing age-related diseases. In behavioral and social sciences, AI is used to streamline and refine coding, uncover latent patterns in large social behavioral datasets, and personalize risk-reduction strategies for older adults. In the health sciences, AI is used to support clinical decision-making, predict adverse outcomes, and enhance patient-provider communication. Scholars in humanities and arts probe philosophical questions like “how should we” and “should we” about humanistic AI models learning to become supportive companions or therapists for older adults, or simulations of deceased loved ones to comfort family members. In social research, policy, and practice, AI-driven models can enhance decision support systems in formal, informal, and self-care settings, as well as service delivery. In higher education, AI offers opportunities to enhance pedagogical practices, including personalizing assignments, customizing learning, creating adaptive learning experiences, assisting with grading, generating classroom content, and synthesizing information for diverse audiences. Emerging scholars are using AI to support their teaching, writing, outlining ideas, and refining data analysis. Panel discussions also underscored shared concerns about AI, including hallucinations, lack of critical thinking and empathy, incorrect or biased information, privacy issues, and equity concerns.

The member groups present a more detailed synthesis below of panelist responses and the ensuing discussion. They highlight the importance of cross-disciplinary convenings to elucidate how this rapidly evolving landscape impacts gerontological researchers and educators and how we can collaborate to produce informed and ethical research and education in this space. Perspectives from the GSA member groups below are presented in alphabetical order but conclude with AGHE and ESPO, given their multidisciplinary representation.

## Disciplinary perspectives

### Biological sciences

Biological sciences in gerontology examine the biological processes that underlie aging, including understanding molecular alterations that feature in the aging process to studying evolutionary forces that determine longevity differences among species. AI-based systems are increasingly being used in the biological sciences across many areas, including identifying genetic markers of aging, predicting biological age, aiding biomarker discovery, and predicting cellular senescence and mitochondrial decline to address age-related diseases. It is crucial to recognize, however, that AI is fundamentally a sophisticated form of statistical pattern matching (Kumar et al., 2023). It does not “think” like a human or possess consciousness, creativity, or genuine understanding. Instead, AI processes large datasets to detect statistical relationships and predict outcomes based on prior observations, thereby enhancing human research while carrying inherent limitations.

AI cannot generate new insights in the same way human scientists can. Whether used in text generation, medical diagnostics, or genetic analysis, AI relies on probabilistic models to forecast the most likely sequence of words or data points. It neither comprehends underlying concepts, forms original hypotheses, nor engages in abstract reasoning. This is particularly relevant in gerontology, where researchers explore fundamental questions about why aging occurs, whether it can be reversed, and how biological systems interact. While AI can detect correlations among longevity genes, protein structures, and cellular aging processes, it is unlikely to independently devise a new theory of aging. Breakthroughs such as Watson and Crick’s DNA structure discovery resulted from creative synthesis rather than mere statistical inference. Similarly, just as an AI trained solely on classical music will never invent jazz, an AI trained on existing aging research can refine models and accelerate discovery but will not create an entirely new aging framework.

Nevertheless, AI’s ability to recognize statistical patterns makes it a potent current and future tool for gerontology (Jumper et al., 2021). In genomics and epigenomics, AI-powered systems identify genetic markers of aging, predict biological age from DNA methylation patterns, and uncover longevity-related pathways. In proteomics and metabolomics, AI assists in identifying age-related changes in proteins and metabolic networks, thereby aiding biomarker discovery even as environmental influences require further validation. Moreover, AI can predict cellular senescence and mitochondrial decline, supporting interventions such as senolytics to remove aged cells and therapies to boost mitochondrial function. Although challenges remain in integrating multi-system data into a coherent model, AI’s capacity to link findings across genes, proteins, and cellular functions underscores its value.

AI’s impact extends beyond research to education, where it personalizes learning experiences and automates aspects of instruction for biological sciences. AI-driven tools help students synthesize research, summarize lectures, and refine their writing. Automated grading systems provide feedback on clarity, argument strength, and accuracy. Additionally, AI can explain complex topics such as the Hallmarks of Aging (López-Otín et al., 2023) at various levels—from non-science majors to graduate students and professionals. By tailoring explanations to user input, AI bridges gaps between introductory and advanced learners, making fundamental aging biology more accessible. It can also generate study materials, produce adaptive quizzes, and assist in structuring research papers. However, as in research, AI enhances rather than replaces the critical thinking and human judgment essential in education.

AI’s strengths come with significant limitations (e.g., ­Sarikaya, 2025). While it excels at statistical pattern matching, it has notable weaknesses: (1) Hallucinations: AI sometimes generates incorrect but highly confident responses. Example: AI may falsely claim, “Albert Einstein was the first person to walk on the moon.” In gerontology, such errors could lead to misleading conclusions in research or medical applications. (2) Lack of Common Sense: AI cannot apply real-world logic. Example: When asked, “I locked myself out of my house. Any ideas?” AI may respond, “Have you tried using your keys?” Such responses highlight AI’s inability to infer situational context, which is crucial in healthcare and aging research. (3) Outdated or Biased Information: AI is only as good as its training data. If trained on incomplete or biased datasets, it may produce misleading conclusions. This is a serious issue in aging research, where biases in demographic data could lead to inequitable health recommendations. (4) Ethical Risks in AI-Driven Biology: AI-generated insights in personalized medicine may prioritize profits over equitable healthcare access. Additionally, the use of AI in synthetic biology raises concerns about privacy violations, genetic discrimination, and ecological risks. These limitations must be addressed to produce accurate, ethical, and equitable information through AI-based systems.

### Behavioral and social sciences

Behavioral and Social Sciences (BSS) within gerontology investigate how individual behaviors, social contexts, and environmental factors interact to influence health, well-being, and quality of life in later life. BSS researchers draw on theoretical frameworks—such as the Stress and Coping Model, Socioemotional Selectivity Theory, and the Life Course Perspective—to understand processes ranging from caregiving dynamics and health‐related decision-making to social engagement and resilience. Methodologically, BSS studies employ mixed methods (e.g., surveys, in-depth interviews, ethnography, longitudinal cohort designs) and advanced statistical modeling (e.g., multilevel modeling, structural equation modeling) to capture both the complexity and the temporality of aging phenomena. Interdisciplinary collaboration—incorporating perspectives from psychology, sociology, public health, economics, and many more—enables BSS researchers to address multifaceted questions about policy, program evaluation, and intervention development.

AI’s emergence intersects with every facet of BSS work: natural language processing can streamline qualitative coding; machine learning can uncover latent patterns in large social‐behavioral datasets; and predictive algorithms can personalize risk‐reduction strategies for older adults. At the same time, AI challenges core BSS concerns—such as maintaining the ecological validity of social interactions, preserving participant privacy, and ensuring equitable access—underscoring the need for thoughtful integration of AI tools into BSS research and practice.

AI is fundamentally reshaping aging research and gerontological education in this area, too, prompting both existential questions and exciting opportunities. At first glance, generative AI tools like ChatGPT appear to have rendered traditional long-term research projects obsolete. For instance, a pilot study evaluated ChatGPT’s capability to provide tailored responses to dementia caregivers and found that it performed at a high level—effectively accomplishing what NIH R01 grant proposals envisioned as 5-year endeavors (Aguirre et al., 2024). This realization led to an unsettling thought: if AI can already deliver these outcomes, does that mean researchers in our field are on the brink of obsolescence?

However, a deeper analysis reveals a more nuanced perspective. While current generative AI tools have achieved impressive milestones, they are not without limitations. In this pilot study, the researchers identified several weaknesses—including inconsistencies in the empathy conveyed, occasional inaccuracies in the details provided, and difficulty in addressing highly nuanced or context-specific queries—that highlight clear gaps where further research is needed. Rather than signaling the end of our roles, these limitations provide a roadmap for new research directions. Researchers can turn this challenge into an opportunity by focusing on improving AI performance, addressing its shortcomings, and tailoring it to the unique needs of aging and caregiving.

The rapid evolution of AI means that the current limitations may soon be resolved by the very systems we are critiquing. This dynamic environment necessitates that we, as researchers, continually adapt our focus and expand our own AI literacy and skills. Staying updated on AI’s evolving capabilities and constraints is essential—not only to refine our research agendas but also to maintain relevance and effectiveness in our roles. In essence, AI literacy becomes the cornerstone that enables us to leverage new technologies while critically evaluating and improving them. It is equally important to recognize that achieving these advancements requires robust interdisciplinary collaboration; this pilot study and larger NIH project brought together dementia care clinicians, information scientists, NLP/ML experts, and social and behavioral researchers, underscoring that lifelong learning and cross-disciplinary cooperation are vital even for those specializing in AI development.

Ethical considerations are equally pivotal. Although privacy and data security are frequently cited concerns, the practical reality for many caregivers may be different. When facing the promise of tailored, high-quality support, caregivers might willingly share detailed personal information—even if it increases their privacy risks. Here, the solution lies in enhancing AI literacy across all parties. Educating caregivers—and the general public—about the inherent risks of data sharing with AI tools facilitates informed decisions. Moreover, the debate between proprietary versus open-source large language models illustrates a fundamental trade-off: superior performance often comes with higher privacy risks. In contrast, open-source alternatives offer more control and transparency at the cost of current performance gaps. As these models evolve, the balance may shift, necessitating ongoing literacy and informed trade-offs.

Ultimately, the future of aging research in the era of AI rests on our ability to stay informed and adaptive. By embracing AI literacy, we safeguard our roles as researchers and ensure that AI is harnessed responsibly to enhance both scientific discovery and caregiving practices.

### Health sciences

Health sciences is an interdisciplinary field that addresses human health, disease, and healthcare and includes physicians, dentists, nurses, pharmacists, nutritionists, and other allied health professionals and researchers in these fields. Health sciences research and practice are rapidly transforming by integrating AI, with implications for clinical care, diagnostics, population health, and system-level operations. AI is increasingly used to support clinical decision-making, predict adverse outcomes, and enhance patient-provider communication, particularly for complex, multimorbid older adults who may otherwise be poorly served by conventional approaches (Abadir & Chellappa, 2024). These developments hold particular promise for more personalized, anticipatory care across care settings while supporting older adults to remain safely and independently in their communities (Abadir et al., 2023).

AI in clinical health sciences spans multiple use cases. Predictive analytics tools, such as mortality risk algorithms, have been shown to increase advance care planning conversations, identify high-risk patients for earlier palliative interventions, and reduce disparities in end-of-life discussions across racial and ethnic groups (Avati et al., 2018; Parikh et al., 2019). These tools exemplify how AI can address long-standing gaps in aging care by helping clinicians identify needs earlier and engage in timely decision-making. Similarly, AI-based tools are increasingly aiding clinicians in day-to-day interactions, for example, by reviewing patient messages, assisting with documentation, or preparing response drafts based on prior visit data—thereby improving efficiency and continuity of care (Abadir & Chellappa, 2024).

Clinical decision support systems have also benefited from AI, especially in imaging and diagnostics. Some AI-enabled surgical decision tools outperform traditional clinical indicators in identifying candidates for complex interventions (De Silva, 2020). AI models have also shown superior performance to radiologists in certain tasks, including early lung cancer detection, prompting more FDA approvals of AI-powered diagnostics (Mathew et al., 2020). Yet, translating these innovations into routine geriatric care still faces barriers, including generalizability, validation in diverse populations, and alignment with person-centered care.

Despite the promise, the integration of AI into health sciences raises unresolved ethical and equity concerns. As with other disciplines, AI models in clinical settings may perpetuate racial, gender, and age biases embedded in training data or deployment environments (Obermeyer et al., 2019; Turner Lee, 2018). These risks are especially salient for older adults, whose care often occurs across fragmented systems and whose voices have historically been excluded from digital technology development. For example, many existing voice assistants and health interfaces are not optimized for older users, and default assumptions about aging-as-disability can further marginalize those who are cognitively or physically intact (Knowles et al., 2021). Addressing these gaps requires full participation of older adults in the co-design of AI tools and increased representation in datasets used to train models (e.g., Cho et al., 2025). Health sciences researchers, developers, and policymakers must remain vigilant in examining who benefits from AI deployment and whose needs may be overlooked.

As with other disciplines, AI models in clinical settings may perpetuate racial, gender, and age biases embedded in training data or deployment environments (Obermeyer et al., 2019; Turner Lee, 2018). In addition to systemic biases, new work shows that AI models themselves may also be vulnerable to cognitive biases—such as confirmation bias and recency bias—that affect clinical reasoning. In a recent evaluation of large language models in medicine, Schmidgall et al. (2024) demonstrated that biased prompts led to significant declines in diagnostic accuracy, with some models showing up to 26% performance degradation. Even GPT-4, the top-performing model, exhibited reduced accuracy under biased conditions. Mitigation strategies—such as bias warnings or reasoning prompts—improved but did not eliminate these effects. These findings underscore that AI systems, like human clinicians, can absorb and express subtle decision-making biases and must be rigorously evaluated before clinical deployment.

As in other GSA member sections, human engagement remains essential in the health sciences. While AI can improve detection, prediction, and administrative efficiency, it does not replicate empathy, judgment, or the holistic understanding of the person-in-context that underpins quality geriatric care. Responsible use of AI must support, not substitute for, clinical expertise, and be deployed within ethical frameworks that account for vulnerability, informed consent, and shared decision-making. Ensuring equitable access to these tools and avoiding “techno-solutionism” will be key as health sciences continue to evolve alongside rapidly advancing AI capabilities.

### Humanities and arts

Humanities and arts scholarship in gerontology focuses on representations and lived experiences of aging and caregiving. It includes disciplines such as medical anthropology, environmental gerontology, social gerontology, social work, clinical psychology, and nursing. Scholars of literature, philosophy, and other humanities disciplines collaborate with research gerontologists to explore what it means to be human in later life, with attention to normative (values-based) considerations about what makes a good life (flourishing, wellbeing) and what social conditions support or hinder flourishing in later life or for caregivers (Berlinger et al., 2022). Critical humanities scholarship draws attention to evolving or problematic aging concepts, especially those that reduce aging to health status or a problem to be solved (Grenier et al., 2017).

There are two big sets of normative questions concerning AI and aging: “how should we?”; and “should we?” The “how should we?” questions, often termed “AI ethics,” are procedural questions centering on safety and transparency. Relevant issues include informed consent and data protection during piloting and deployment, evaluation, and regulation. For example, when AI models are being piloted in patient or client contexts, how transparent should these activities be? Examples of normative scenarios now frequently arising in aging-related care include AI, or ambient, scribes for notetaking during conversations with patients and caregivers, and AI models to reduce clinical uncertainty in treatment recommendations (Goodman et al., 2024; Lukac et al., 2025; Trang, 2025). These real-world scenarios necessitate clear recognition of patient vulnerabilities and attention to how thoughtful use of AI models by practitioners can lead to better representation of the voices and perspectives of older adults and caregivers in electronic health records. Normative concerns also include the risk of ceding practical skills, such as communication and clinical judgment, to AIs and other technologies (Vallor, 2011). These concerns are especially relevant in gerontology, given older adults’ frequent interactions with healthcare systems to treat aging-associated health conditions.

The “should we?” questions concern AI as a rapidly evolving social phenomenon. These questions are less obviously about research conduct and more about the role of AI in human life, including its role as a substitute for human presence and capacities. Humanistic questions arise throughout a person’s life, about what matters, what makes a good life, and how relationships, environments, and experiences shape an individual’s sense of what is meaningful and valued. Understanding how AI and other technologies can enhance or undermine our humanity are crucial questions for aging and for caregiving. Beyond the hype about transformative AI models, there is much we don’t yet know about the human-AI relationship (Binkley & Pilkington, 2023). We do know that humans are susceptible to falling in love with technology itself, and AI models designed to simulate human communication through tone and voice may be especially likely to promote attachment (Cherelus, 2024). We know that AI models can learn to engage humans by being supportive companions, therapists, and even simulations of a loved one who has died (Hwang et al., 2024; Jecker, 2024; Misselhorn, 2023; Yang, 2024). Chatbots have been shown to outperform humans in some professional roles, and some ­individuals, when given a choice, will prefer to interact with a humanistic chatbot rather than a human professional (Goh et al., 2025; Hswen & Rubin, 2025).

We need much more research, thoughtful piloting, and practice on the humanistic questions that these findings reveal. What are we willing to cede to AI models, and what should we keep for ourselves as human capacities (Vallor, 2024)? Can co-designing AI with older adults support flourishing by extending everyday capacities (Chan et al., 2024)? If so, who will have access to these tools, and what support do they need to use them (Czaja et al., 2024)? Are monitoring devices and chatbot friends good-enough replacements for human presence (Broadbent et al., 2024; Intuition Robotics, 2024)?

Thinking about the intertwined social phenomena of AI and population aging engages values and ideas about the future that inevitably reflect concerns about the present. In aging societies with too few human caregivers and ever-escalating care costs, will human connection become a luxury for the elite, with carebots for everyone else? As we stretch the limits of 20th century “robot” metaphors in imagining our present and future, humanities scholarship and creative work in the arts (Superflux, 2021) can include useful and engaging ways of thinking together about these challenges.

### Social research, policy, and practice

The Social Research, Policy, and Practice group of GSA is committed to improving policies and services for older adults and their families and includes practitioners and researchers in social work, social policy, nursing, economics, sociology, political science, medicine, planning, pastoral counseling, and business. Advances in AI are driving important lines of research in this area while raising important questions that must be addressed.

AI-driven models promise to improve decision support systems in formal, informal, and self-care. Model-based clinical decision support systems have a long history, but improvements and new applications are proposed as more sophisticated modeling techniques and data types become available (Bozyel et al., 2024; Zheng et al., 2024). Advances in the affordability and usability of technology have increased the opportunity to test the deployment of AI models to support informal caregiving and self-care activities. Example applications include providing automated advice to informal caregivers of persons living with dementia, evaluating the accuracy of model-generated facts about epilepsy, and personalized physical activity recommendations based on real-time user context (Hasan et al., 2024; Kim et al., 2024; Sun et al., 2024). Research in this area focuses on improving model performance and technology accessibility and building evidence around technology’s impact. A key open problem is who bears the responsibility for incorrect recommendations provided by advanced AI and, in the clinical setting, who holds decision-making authority (Funer et al., 2023).

A related application of AI receiving attention is the deployment of advanced models to use complex data from multiple sources or collected continuously to monitor health and diagnose health problems (Alexandru et al., 2024; Darwish, 2024; Husnain et al., 2024; Yu et al., 2024; Zhou et al., 2024). Although designed to be more autonomous than traditional clinical decision support systems, this application also raises the issue of how best to train clinicians or other users to interact with this technology such that it complements but does not replace their expertise.

Automated creation or curation of data is another promising area for applying AI. Examples include the creation of variables from clinical notes, identifying sentiment from service reviews, and even generating synthetic healthcare-related training images that are not subject to data security laws (Ching et al., 2025; Cunningham et al., 2024; Hotchkiss et al., 2024; Zaidat et al., 2024). Much of the work in this area focuses on validating method performance. The increased use of automation raises the importance of evaluating algorithms for bias (Mamo et al., 2024).

Not quite as far along in the research pipeline are ideas around automation of service delivery, such as drone-delivered medication, autonomous vehicles providing non-emergency medical transport, and robots assisting in the home (Leon et al., 2025; Nithila et al., 2025; Silvera-Tawil, 2024). Work in this area is at the level of understanding user acceptability and trust and the scalability and logistics of these technologies. AI shows promise for improving service delivery, but policies and standards around decision-making authority, culpability, user training, and algorithm fairness are needed.

### Academy for Gerontology in Higher Education

The Academy for Gerontology in Higher Education (AGHE) member group in GSA includes educators across disciplines who teach gerontology or geriatrics courses in higher education, including two-year and four-year colleges, universities and professional schools, and students interested in teaching. Ensuring a well-prepared workforce capable of addressing the complex and evolving needs of our aging population is the responsibility of institutions of higher education throughout the world. In these training environments for future gerontologists and aging specialists, educators aim to impart the interdisciplinary knowledge necessary to understand and address the diverse experiences, needs, and contributions of older adults within societal frameworks. Through this knowledge exchange, we aim to not only prepare the workforce but also to continue the development of gerontological theory and practice. ­Gerontology education also contributes to the advancement of pedagogical techniques that reflect the interdisciplinary, lifespan-oriented nature of the field. By integrating innovative teaching strategies, experiential learning, and responsive curricula, gerontological education not only equips students with essential competencies but also fosters learning environments. These dual aims, strengthening the aging services workforce and enhancing teaching practices, are central to promoting both professional readiness and academic rigor in the field. As such, the rise of readily available artificial intelligence is of great concern, as it affects how our students may develop critical thinking, engage with course material, and demonstrate original thought, potentially challenging traditional pedagogical approaches, necessitating the reevaluation of assessment strategies to ensure academic integrity and meaningful learning.

Concurrently, using generalized large language models and machine learning is not new to gerontological education. One must only look at the near prevalence of spellcheck tools for written documents and the use of grade books in learning management systems, to name a few, to see how these models have been widely used in education for decades. However, the widespread accessibility of AI to consumers has prompted higher education institutions to critically examine their pedagogical practices and learning expectations (Aler Tubella et al., 2024). This was partly because the rapid launch and uptake of these tools were shocking to a large percentage of the population who were not intimately familiar with their development. They represented a sudden change to the status quo, to which higher education was initially slow to respond.

While initial concerns regarding an “AI Apocalypse” in academia have not materialized, AI has nonetheless necessitated reevaluating how courses and assignments are structured (Crompton & Burke, 2023). AI presents significant opportunities for enhancing pedagogical practices. For instance, educators can use AI to design customized avatars and case studies, enabling students to engage with personalized assignments tailored to their interests. This capacity for individualized learning fosters a more student-centered approach, facilitating the development of flipped classrooms and adaptive learning environments.

Beyond curriculum design, AI has also assumed various administrative responsibilities within teaching, such as grading discussion boards and evaluating structured assignments with clear parameters. We argue that by automating these routine tasks, AI allows instructors to cultivate meaningful one-on-one relationships with students. Additionally, AI is a valuable tool for content development, assisting in creating presentations and synthesizing information for diverse audiences.

One of the most promising applications of AI in higher education is its ability to support dynamic, adaptive learning experiences. AI-driven course design can modify instructional content based on student performance in real time. For example, if one student excels on a quiz while another struggles, AI can adjust subsequent learning modules to reinforce concepts for the latter while allowing the former to progress to more advanced material. This adaptability enhances opportunities for deeper engagement with course content, particularly in areas where time constraints might otherwise limit exploration.

However, as AI becomes increasingly integrated into educational settings, institutions must also navigate the evolving expectations surrounding human connection in learning. While AI offers valuable tools for personalization and efficiency, maintaining meaningful interpersonal interactions remains essential to fostering an enriching educational experience. Within gerontological education, it is critical to recognize the existing ageist bias that may be present in many models, particularly when it is often unclear what and how they were trained. Furthermore, instructors must remember that AI is a tool lacking the pedagogical training and theoretical backing to design effective learning independent of the content expert.

While an initial shock to the status quo, higher education is now beginning to embrace these AI models for the tools and resources they provide. As educators, we must work to prepare our students for the careers they will enter, which will increasingly involve AI applications.

### Emerging scholars

The Emerging Scholar and Professional Organization (ESPO) member group spans the disciplinary member groups highlighted above and supports undergraduate, graduate, and transitional (i.e., postdoctoral, early-career, and pre-tenure faculty) members through professional development, writing support, and networking. Regardless of the disciplinary lens through which we approach our work, emerging scholars and professionals in gerontology are united in our reality that AI is inextricably linked to the future of research, education, and practice. Unlike previous generations, we are entering our academic and professional careers in a landscape where AI is ever-present, simultaneously offering promise and challenges (Barros et al., 2023; Jiang et al., 2024; Rawas & AlSaeed, 2024). The rapid pace of AI development means that new tools are constantly emerging, requiring continuous adaptation and adoption of a growth mindset in relation to AI—a recognition that we do not yet fully understand its implications and must remain open to learning and evolving with the technology ­(Barros et al., 2023; Rawas & AlSaeed, 2024; Watkins, 2025). This leads us to enter AI discussions with more questions than answers, particularly around effective and responsible uses, as this innovation proves to be a facet of our professional lives that is here to stay. This section describes the ways in which AI is shaping research and education, as well as the ethical implications of its use and advancement from the perspective of emerging scholars.

Informational polling and informal conversations among early-career GSA members reveal a range of AI engagement, from daily use to complete avoidance. Like those in other fields, emerging scholars studying aging use AI for practical components of their work, including outlining ideas, assisting with writing (especially early drafting and editing), generating examples to illustrate concepts, developing lecture materials, and refining code (Jiang et al., 2024; Lund et al., 2023; Renkema & Tursunbayeva, 2024). Early-career users in our networks lauded AI for its efficiency, assistance in overcoming creative blocks, and ease of use. However, barriers to entry remain, highlighting the need for training around optimizing prompts, fact-checking AI-generated content, engaging critically, and strengthening other skills that transfer beyond today’s AI capabilities (Jiang et al., 2024; Watkins, 2025). Emerging scholars also expressed more abstract concerns and confusion around ethical standards as the ubiquity of AI in classrooms and workplaces rises. For example, agreed-upon guidelines for handling plagiarism and intellectual property are of paramount importance yet have not been definitively established (Lund et al., 2023); AI-generated content blurs the lines of originality and ownership in ways that early-career scholars must consider.

AI is shaping gerontological research and education in profound ways. While we have historically integrated technology within academia—from calculators to spell check to statistical software—AI presents new, complex challenges that require tactful navigation (Barros et al., 2023; Rawas & AlSaeed, 2024; Renkema & Tursunbayeva, 2024; Watkins, 2025). Academic integrity remains a key issue, as does ensuring that AI use does not reinforce inequities in research, education, and practice (Jiang et al., 2024; Lund et al., 2023); there are valid concerns that scholars who do not engage with AI risk being left behind in a competitive academic atmosphere (Renkema & Tursunbayeva, 2024). Therefore, graduate programs, departments, and professional organizations should ensure students and faculty have the knowledge and skills to navigate AI effectively (Barros et al., 2023). Ultimately, AI has a place in gerontological research, education, and practice; we are responsible for using it with intention and integrity. As we continue exploring its role throughout the remainder of our careers, we—as emerging scholars and professionals—must remain open and cautious, embracing AI as a tool while critically examining broader implications.

## Discussion

While AI is shaping research and education differently across disciplinary spaces, important overlapping questions, concerns, and opportunities become more visible with cross-disciplinary discussion and collaboration, including strategies for navigating ethical quandaries and equity concerns. One key takeaway from this session was that AI technology still requires human engagement across disciplines. Despite immense opportunities, AI cannot replace interpersonal human connections, engage in abstract reasoning, or generate new creative insights that move beyond statistical pattern matching. In its current form, AI lacks empathy, is often riddled with inaccuracies, and poses challenges related to privacy and data security that humans can help offset in various ways. However, some older adults and care partners may be willing to forgo concerns about these challenges to access certain health interventions or other tools. This leads to two more significant questions posed explicitly and implicitly across disciplines about *how* we should use AI and *whether* we should use AI at all in certain circumstances. These are philosophical questions with policy implications that humans must grapple with in the context of evolving AI-related technologies, and cross-disciplinary collaboration can provide invaluable insights to addressing these questions.

A second key takeaway is that across disciplines, experts emphasized how using AI tools can provide individualized support for older adults, care partners, and gerontology students. For example, AI tools in education can provide adaptive learning experiences that can modify instructional content based on student performance in real time. AI tools can also improve decision-making and support systems in care, health monitoring, and diagnostics based on individual health data, and provide customized interventions to support patients and caregivers.

A third common takeaway from this multidisciplinary session was the importance of training as a strategy to increase knowledge and address concerns about ethical implementation and equity when using AI-based systems. Many gerontological researchers, educators, and practitioners are new to AI tools. The rapid pace of development requires critical inquiry and training on diverse uses and implementation of AI, ethics, and equity considerations. Training must also incorporate a related theme that emerged in this session: adaptability. Humans using AI must be willing and able to adapt to meet the evolving landscape of AI technologies in aging. Any policies developed around AI should incorporate space for training that allows for human adaptation. We argue that cross-disciplinary conversations can build robust training modules that more comprehensively examine opportunities and concerns about AI use, including ethical implementation and equity.

Additionally, concerns about bias arose across disciplines. Increasing use of AI for clinical decision support, predictive analytics, and diagnostics in aging care provide clear benefits in identifying at-risk patients and enhancing care planning but must be developed and deployed with caution. As recent studies have shown, even the most advanced large language models are susceptible to cognitive biases—such as confirmation and recency bias—that can skew diagnostic outputs (Schmidgall et al., 2024). Algorithmic fairness, inclusive design, and evaluation frameworks that include older adults as partners are essential to mitigate harm and promote equity. Clinical AI must augment—not replace—professional judgment, and its integration should be guided by both scientific rigor and ethical ­vigilance to address bias. Cross-disciplinary collaboration can help identify solutions for addressing bias that may otherwise be overlooked through a narrower disciplinary lens.

Finally, experts across disciplinary spaces emphasized common challenges around accuracy, privacy, and security that demand further cross-disciplinary conversations and collaborations. Hallucinations and inaccuracies continue to plague emerging AI technologies in gerontological research, practice, and education. Privacy and data security also present real challenges that scholars, practitioners, and students in aging must address. While some AI technologies may provide improved accuracy for certain clinical diagnoses compared to screenings or human clinicians, these benefits must be balanced with the very real drawbacks (e.g., bias, data security) that continue to exist. Older adults, care partners, and practitioners may also make different calculations to balance competing interests relating to AI technologies that may be informed by certain disciplinary training and experience. More disciplinary collaboration is needed to solve some of these persistent thorny issues.

While this article summarizes diverse disciplinary insights from the six GSA member groups at GSA’s annual conference, it by no means reflects a comprehensive overview of disciplinary perspectives on this issue. We acknowledge that many perspectives are not reflected, and more research and conversations are needed that include these perspectives (e.g., feminist; critical race theory; science, technology, and society). However, through this GSA session and paper, we hope to underscore the importance of multidisciplinary perspectives and encourage more cross-disciplinary scholarship and conversation in emerging research and policy discussions about AI and aging.

## Conclusion

This multidisciplinary conversation presented several ideas for moving forward that are relevant for all the GSA member groups and disciplinary orientations. First, researchers and educators in aging must be ready to adapt to and stay informed of the evolving landscape of AI. This does not mean compromising on important ethical questions surrounding AI but instead means that gerontologists and geriatricians need to be at the table, informing research, practice, and policy on aging that is shaped by the growing presence and use of AI. Second, researchers and educators in aging should proactively address ageism and other biases that currently plague AI. This could involve incorporating older adults into research or educational practices through co-design or other avenues that elevate the voices and lived experiences of older adults. Ultimately, researchers and educators in aging must recognize that human engagement is crucial to our work, including projects involving AI. It cannot engage in critical thinking, infer situational context, or express empathy. These are skills that no predictive algorithms or statistical pattern matching can accomplish—at least not yet—and are necessary to support responsible, ethical, and accurate outcomes when using AI. Ultimately, by sharing information and understanding the myriad ways that AI technologies are evolving and informing various interrelated disciplinary spaces, we can create policies and practices that ensure that gerontological students, researchers, and practitioners can effectively, efficiently, and ethically use AI technologies.

## Data Availability

This article does not report data and therefore the pre-registration and data availability requirements are not applicable.
